# A pocket practical guide on bibliometric analysis: bridging informatics with science in a rapid manner

**DOI:** 10.1097/MS9.0000000000002436

**Published:** 2024-08-08

**Authors:** Huiyang Li, Haixiao Wu, Chao Zhang

**Affiliations:** a Department of Gynecology and Obstetrics, Tianjin Medical University General Hospital; b Department of Bone and Soft Tissue Tumor, Tianjin Medical University Cancer Institute and Hospital, National Clinical Research Center for Cancer, Key Laboratory of Cancer Prevention and Therapy, Tianjin’s Clinical Research Center for Cancer, Tianjin, People’s Republic of China

*Dear Editor,*

We recently read with great interest the article by Zhang *et al*.^1^ published in the *International Journal of Surgery*. The study summarized the surgical simulation training and learning curve using bibliometric analysis (BA). The results can guide readers in understanding the current state and future trends in the surgical education field. After wide works of literature reviews, we found several latest BA papers published in the *International Journal of Surgery* ^2–4^. Being a rigorous strategy for exploring large volumes of literature, BA was used to unpack the evolutionary nuances and research trends of the specific topic. The emerging areas and the future direction of the field can be revealed. Several suggestions on performing BA have been recently reported; however, a practical guide is needed^5^. In order to bridge health informatics with medical science in a rapid manner, we presented the practical guide on BA (Fig. 1). Herein, the guide was explained step by step.

**Figure 1:**
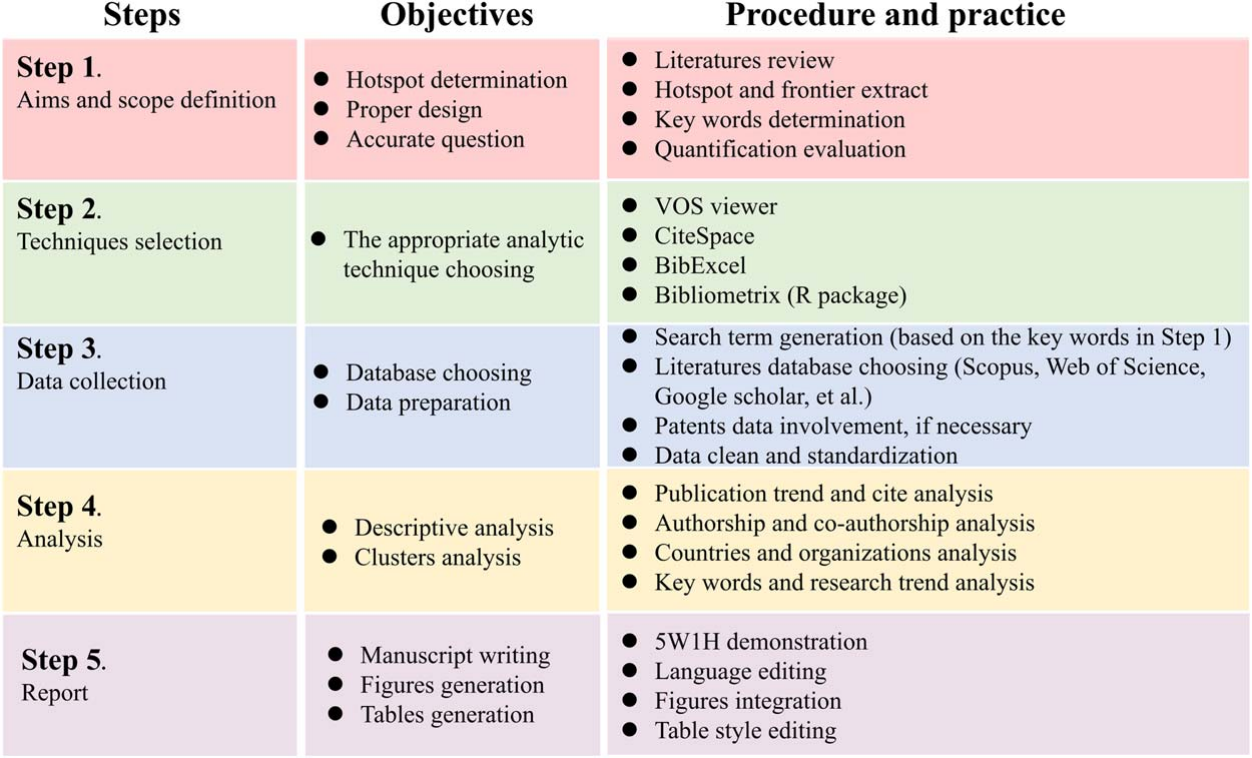
The pocket practical guide on bibliometric analysis.

## Step 1. Aims and scope definition

The topic of interest should be primarily determined. The accurate scientific questions from the specific field are the guarantee for a high-quality BA study. The aims of the BA study can be set out to unpack the mass research constituents and to reveal the networks. The scope definition can be determined based on the publication amount. Considering the adequate data for comprehensive analysis, the publications on the topic should exceed 500.

## Step 2. Techniques selection

A total of four common techniques were used, including VOSviewer, CiteSpace, BibExcel, and Bibliometrix. VOSviewer, being an open-source software, was widely used for network analysis. CiteSpace can visualize the research trend. The advanced customization options can improve the accuracy and visualization. As an efficient analysis technique on BibTex and EndNote, BibExcel showed its prominent ability on visualizing networks. Bibliometrix, being an R package, is adept at handling mass data extraction and analysis. The combination of the techniques can make BA comprehensive and informative. Users can choose their technique based on scientific questions, data types, computer configuration, and their working background.

## Step 3. Data collection

To collect publications, the researchers should determine the database. Various databases were previously used, including Web of Science (WoS), Scopus, PubMed, and Dimension. WoS and Scopus are widely used databases that provide comprehensive works of literature information, such as year of publication, citation, authorships, and key words. The research funding and patent information are available on the Dimension platform. With the confirmed topic from Step 1, the researchers can use multi-databases and need to combine the data from multi-databases into a cleaned and standardized format.

## Step 4. Analysis

The analysis step can be divided into the descriptive analysis and the cluster analysis. The descriptive analysis is performed based on the general information, including publication trend, citation analysis, journal analysis, and country and organization summarization. Such descriptive results, including publication growth, productivity, and journal impact, can reveal the popularity of the direction and provide the dynamics of the topic. The cluster analysis is performed based on the extracted data, including authorships, collaborations, key words, and citation bursts. The thematic and/or social clusters can be generated, which can promote the understanding of the development and the structure of the specific topic.

## Step 5. Report

Combined with the data unpack and results interpretation, the core value of BA lies in the discussion section. Researchers can organize the discussion section based on the ‘5W1H’ method, namely What, Where, When, Who, Why, and How. This method can help report generation in a clear and rapid manner.

## Ethical approval

Ethics approval was not required for this correspondence.

## Consent

Informed consent was not required for this correspondence.

## Source of funding

This work was supported by the Key Laboratory of Tumor Immunology and Pathology (Army Medical University), the Ministry of Education (2021jsz704) and the Natural Science Foundation of China (81702161, 82011530050).

## Author contribution

H.L. and H.W.: searched works of literature and wrote the draft; C.Z.: revised the final manuscript. All authors approved the submission of the manuscript.

## Conflicts of interest disclosure

The authors have no conflicts of interest.

## Research registration unique identifying number (UIN)

Not applicable.

## Guarantor

Chao Zhang, the corresponding author of the manuscript.

## Data availability statement

Data sharing is not applicable to this article as no new data were created or analyzed in this study.

## Provenance and peer review

The submitted paper was a correspondence, it was not invited.
